# Positive Modulation of Pink *Nelumbo nucifera* Flowers on Memory Impairment, Brain Damage, and Biochemical Profiles in Restraint Rats

**DOI:** 10.1155/2016/5789857

**Published:** 2015-12-08

**Authors:** Thawatchai Prabsattroo, Jintanaporn Wattanathorn, Pichet Somsapt, Opass Sritragool

**Affiliations:** ^1^Department of Physiology and Graduate School (Neuroscience Program), Faculty of Medicine, Khon Kaen University, Khon Kaen 40002, Thailand; ^2^Integrative Complementary and Alternative Medicine Research and Development Center, Khon Kaen University, Khon Kaen 40002, Thailand; ^3^Department of Physiology, Faculty of Medicine, Khon Kaen University, Khon Kaen 40002, Thailand; ^4^Division of Nuclear Medicine, Department of Radiology, Faculty of Medicine, Khon Kaen University, Khon Kaen 40000, Thailand

## Abstract

Due to the crucial role of oxidative stress in the stress-induced memory deficit, the benefit of substance possessing antioxidant effect is focused. Since no data are available, we aimed to determine the effect of *Nelumbo nucifera* flowers extract on spatial memory and hippocampal damage in stressed rats. Male Wistar rats, weighing 250–350 g, were orally given *N. nucifera* extract at doses of 10, 10, and 200 mg·kg^−1^ 45 minutes before the exposure to 12-hour restraint stress. The spatial memory and serum corticosterone were assessed at 7 and 14 days of study period. At the end of study, acetylcholinesterase (AChE), monoamine oxidase type A and monoamine oxidase type B (MAO-A and MAO-B), oxidative stress status, neuron density, and Ki67 expression in hippocampus were also assessed. The results showed that *N. nucifera* extract decreased memory deficit and brain damage, serum corticosterone, oxidative stress status, AChE, and MAO-A and MAO-B activities but increased neuron density and Ki67 expression in hippocampus. These suggested that the improved oxidative stress status, adult neurogenesis, and cholinergic and monoaminergic functions might be responsible for the protective effect against stress-related brain damage and dysfunction of the extract. Therefore, *N. nucifera* extract is the potential neuroprotective and memory enhancing agent. However, further researches are still required.

## 1. Introduction

Daily stress produces a great burden on neuropsychological problems including cognitive impairment. Stress can stimulate the responses via the hypothalamic-pituitary-adrenocortical (HPA) axis and the sympathetic-adrenomedullary (SAM) system. It has been reported that stress increases the monoaminergic system functions with a subsequent increase in oxidative stresses [1] and memory impairment [2]. In addition, the stimulation of HPA axis induced by stresses also gives rise to the elevation of glucocorticoids (GCs) leading to dendritic atrophy and neurodegeneration in the CA3 region together with the suppression of neurogenesis in dentate gyrus of hippocampus [3]. It has been reported that the prevalence of memory impairment is increased in company with the increased daily stress exposure. Unfortunately, no current drugs can prevent brain damage and memory impairment induced by stress. Therefore, effective neuroprotective agent against this condition is required. A substantial evidence has demonstrated that substances possessing antioxidant activity can prevent the brain damage [4–6] and memory deficit [4, 7] induced by stress. Therefore, the protective effect against stress-related brain damage and dysfunctions of medicinal plant possessing antioxidant activity has been considered.

*Nelumbo nucifera* Gaertn or sacred water lotus is belonging to the family of Nymphaeaceae. It is widely used in the indigenous system of medicine of many countries. It is also used as health beverages for treating hypertension, cancer, diarrhea, fever, weakness, infection, and body heat imbalance [8].* In vitro* data show that the flower extract of* N. nucifera* exhibits antioxidant activity [9]. Based on its antioxidant effect, we hypothesized that the flower extract of* N. nucifera* might mitigate brain damage and memory impairment induced by stress. Therefore, we aimed to determine the protective effect of* N. nucifera* flower extract on memory and hippocampal damage. The possible underlying mechanism was also investigated.

## 2. Materials and Methods

### 2.1. Plant Materials and Preparation

**Figure 1:**
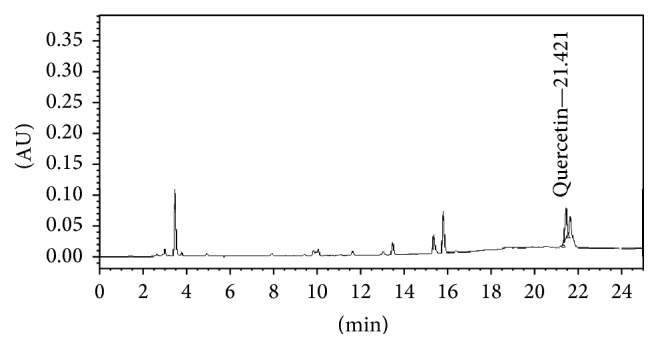
The fingerprint chromatogram of 50% hydroalcoholic extract of* Nelumbo nucifera* flowers used in this study.

Flowers of* N. nucifera* were harvested during November-December 2012 from Khon Kaen province, Thailand.* N. nucifera* were authenticated by Dr. Nopachai Chansilp, Rajamangala University of* Technology* Tawan-ok. The herbarium specimen was deposited at Integrative Complementary Alternative Medicine Research and Development Center, Khon Kaen University, after the authentication. The flowers were cleaned, cut into small pieces, dried in oven at 40°C, and ground into powder. Then, the powder was extracted with 50% hydroalcoholic solvent by maceration technique and filtered through Whatman filter paper number 1. The filtrate was concentrated with rotator evaporator at 45°C and kept at 4°C until being used. The percentage yield of the extract was 10.23%. The finger print chromatogram of* N. nucifera* showed that the total phenolic compounds and quercetin concentrations were 152.963 ± 0.009 mg GAE·g^−1^ extract and 0.456 ± 0.0001 mg QE·mg^−1^ extract, respectively, as shown in Figure 1.

### 2.2. Animal Treatment

Adult male Wistar rats, weighing 250–350 g, were purchased from National Laboratory Animal Center, Salaya, Nakhon Pathom province. They were housed, six per cage, under standard conditions and maintained under a 12 : 12 dark-light cycle. Temperature was controlled at 24 ± 1°C. Food and water were available* ad libitum* throughout the experiments.

### 2.3. Experimental Protocol

Male rats at the age of 8–12 weeks were trained for learning and memory for 7 days. The trained animals were divided into various groups (*n* = 6/group) as described below.

*Group I*. It is naïve control (nontreated group).

*Group II*. Vehicle plus stress: rats were orally given distilled water 45 minutes before being subjected to 12 h restraint stress exposure.

*Group III*. Tianeptine: rats were orally given Tianeptine at dose of 15 mg·kg^−1^ 45 minutes before being subjected to 12 h restraint stress exposure. (Tianeptine was previously shown to improve memory impairment induced by stress [10] so it was used as positive control.)

*Groups IV–VI*.* N. nucifera* flowers extract treated groups: rats were orally given hydroethanolic extract of* N. nucifera* extract at doses of 10, 100, and 200 mg·kg^−1^ 45 minutes before being subjected to 12 h restraint stress exposure. (These doses were selected based on our pilot data.) 

The assigned treatments were carried out once daily throughout a 14-day study period. The determination of spatial memory and serum corticosterone was performed every 7 days until the end of study period. The biochemical changes including malondialdehyde (MDA) level and the activities of superoxide dismutase (SOD), catalase (CAT), glutathione peroxidase (Glu-Px), acetylcholinesterase (AChE), monoamine oxidase type A (MAO-A) and monoamine oxidase type B (MAO-B), and the expression of Ki67 (adult proliferation marker) together with the histopathological changes in hippocampus were also evaluated at the end of study.

### 2.4. Determination of Spatial Memory

In this part, the animals' spatial memory was assessed by using Morris water maze test. The circular pool (170 cm in diameter × 58 cm in length) was divided into 4 quadrants and filled with water (25°C, 40 cm depth). The water surface was covered with nontoxic milk powder. The removable platform was immersed under the surface water in one of the quadrants. Each rat was trained to memorize the location of an invisible platform by forming the association between its location and the location of a platform by using external cues. The time the animals spent swimming to find the hidden platform was recorded as escape latency. Twenty-four hours later, the animals were reexposed to the same condition except that the platform was removed in order to assess the capability to retrieve and retain information and recoded as retention time.

### 2.5. Corticosterone Assay

Blood was collected and kept on ice at 7 and 14 days of study period. Then, it was centrifuged immediately at 2000 ×g at 4°C for 15 min. The obtained serum was kept at −80°C until being used. Corticosterone levels were measured using Corticosterone Double Antibody Radioimmunoassay Kit (MP Biomedicals). The results were showed as ng/mL.

### 2.6. Determination of Acetylcholine Esterase (AChE) Activity

After the isolation, hippocampus was homogenized with sodium phosphate buffer (0.1 M, pH 7.4) and centrifuged at 14000 ×g at 4°C for 20 min. The supernatant was harvested and used for the determinations of AChE activity and protein concentrations by Ellman et al. [11] and Lowry methods [12], respectively.

### 2.7. Determination of Monoamine Oxidase A and B Activities

The activities of monoamine oxidase type A and monoamine oxidase type B (MAO-A and MAO-B) were evaluated by the continuous peroxidase-linked photometric assay according to the method of Holt et al. [13] with some modifications. Hippocampus was homogenized and centrifuged at 14000 ×g, at 4°C for 20 min. After the centrifugation, the supernatant was harvested for the determination of MAO-A and MAO-B activities. In brief, 40 *μ*L of brain homogenate was incubated with the mixture containing 120 *μ*L amino substrate (2.5 mM tyramine (Sigma-Aldrich) in potassium phosphate buffer), 40 *μ*L chromogenic solution (1 mM vanillic acid, 0.5 mM 4-aminoantipyrine, and 4 U/mL peroxidase in potassium phosphate buffer) for 30 min at 37°C. The 30-minute preincubation of brain homogenate with either pargyline or clorgyline at 37°C was carried out to determine MAO-A or MAO-B activity. The reactions were assessed by using microplate reader at 490 nm.

### 2.8. Determination of Oxidative Stress Markers

Hippocampal homogenate was prepared using sodium phosphate buffer (0.1 M, pH 7.4) and subjected to the 15-minute centrifugation at 4°C for 15 min. Then, the supernatant was collected and used for the determination of oxidative stress markers including MDA level and the activities of SOD, CAT, and GSH-Px. SOD activity was determined via McCord and Fridovich method [14] whereas CAT and GSH-Px activities were determined via the method of Chance and Maehly [15] and the method of Rotruck et al. [16], respectively. SOD, CAT, and GSH-Px activities were expressed as unit·mg^−1^ protein. In addition MDA level was also determined by using thiobarbituric acid (TBA) assay [17] and expressed as nmol·mg^−1^ protein.

### 2.9. Cresyl Violet Staining

At the end of experiment, brains were isolated after the transcardial perfusion with NSS and postfixed with 4% paraformaldehyde in 0.1 M phosphate buffer pH 7.4 at 4°C overnight. Then, they were immersed in cryoprotectant containing 30% sucrose for 48–72 h. Brains were cut at 10 *μ*m thickness and picked up on slides coated with a 0.01% aqueous solution of gelatin. The serial sections were stained with 0.5% cresyl violet. Six coronal sections from each rat in each group were studied quantitatively. Neuronal counts were performed by eye using a 40x magnification with final field 255 *μ*m^2^ and Bregma coordination according to the following stereotaxic coordinates: AP −4.8 mm, lateral ±2.4–6 mm, and depth 3–8 mm. The observer was blinded to the treatment at the time of analysis. Counts were made in five adjacent fields and the mean number extrapolated to give total number of neurons per 255 *μ*m^2^.

### 2.10. Determination of Ki67 by Western Blotting

Hippocampus was isolated and prepared as homogenate by using ice cold RIPA buffer with protease inhibitors. The homogenate was subjected to the 14,000 ×g centrifugation at 4°C for 20 minutes. The supernatant was harvested and we measured the level of protein by using NANO drop Spectrophotometers. Equal amounts of protein (50 *μ*g) were fractionated by 8% SDS polyacrylamide gel electrophoresis and transferred to a polyvinylidene difluoride membrane (Bio-Rad Laboratories, Hercules, CA). Each step was preceded by three times rinsing with the solution containing 0.05% Tris-buffer saline with Tween-20 for 5 minutes each. Membranes were blocked with 5% skim milk in Tris-buffer saline with 0.05% Tween-20 and incubated with the primary antibody against Ki67, a proliferative marker (1 : 500), overnight. After this process, the membranes were incubated with horseradish peroxidase-linked secondary antibody (1 : 4,000) for 1 hour at room temperature. Then the signal was enhanced with a Thermo Scientific Pierce ECL Substrate chemiluminescence kit (Pierce ECL Western Blotting) [18]. Images were acquired by ImageQuant LAS 4000, GE Healthcare. Band densities were quantified with NIH-ImageJ (Version 1.48V; National Institutes of Health, USA). The PVDF was reprobed with the beta actin antibody (1 : 2,000) as a loading control.

### 2.11. Statistical Analysis

Data were expressed as mean ± S.E.M. The significance of differences among the groups was assessed using one-way analysis of variance (ANOVA) test followed by LSD multiple comparison test using SPSS, version 13. In addition, repeated measures ANOVA was used to analyze escape latency and retention time within group at various treatment durations. Moreover, paired *t*-test analysis was implemented on corticosterone level. *P* value < 0.05 was considered as significance.

## 3. Results

### 3.1. Effect of* N. nucifera* Flowers Extract on Spatial Memory

**Figure 2:**
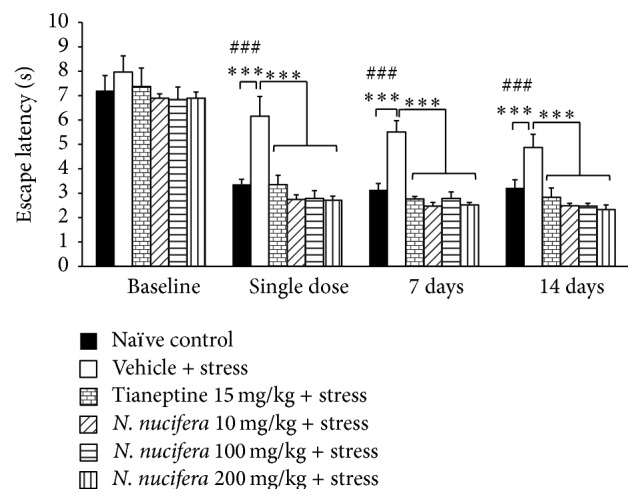
The effect of hydroalcoholic extracts of* N. nucifera* flowers extract on escape latency of stress-exposed rats at baseline, after a single dose, and at 7 days and 14 days of treatment. Data were expressed as mean ± S.E.M. (*n* = 6/group). ^*∗∗∗*^ *P* value < 0.001, compared with vehicle plus stress. ^###^ *P* value < 0.001, respectively, compared with control group.

Figure 2 showed that rats that are subjected to restraint stress and received vehicle or water significantly increased escape latency after a single dose of administration and this change was still observed both at 7 and at 14 days of treatment (*P* value < 0.001 all, compared with naïve control group). Tianeptine attenuated the increased escape latency in restraint stress rats throughout the study period (*P* value < 0.001 all, compared with vehicle + stress treated group). It was found that rats which received* N. nucifera* at all doses and are exposed to 12 h restraint stress significantly decreased escape latency after a single-dose administration and the changes were still observed at 7 and 14 days of treatment (*P* value < 0.001, compared to vehicle + stress). In addition, repeated measures ANOVA was also determined to observe the time effect on escape latency and retention time after restraint stress at the various treatment durations. It was found that no effect of treatment duration on escape latency was observed.

**Figure 3:**
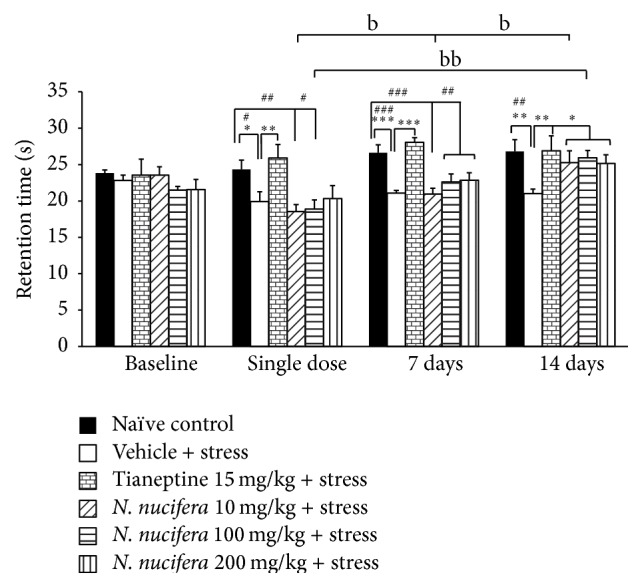
The effect of hydroalcoholic extracts of* N. nucifera* flowers extract on retention time of stress-exposed rats at baseline, after a single dose, and at 7 days and 14 days of treatment. Data were expressed as mean ± S.E.M. (*n* = 6/group). ^*∗*, *∗∗*, *∗∗∗*^ *P* value < 0.05, 0.01, and 0.001, respectively, compared with vehicle plus stress. ^#, ##, ###^ *P* value < 0.05, 0.01, and 0.001, respectively, compared with control group. ^b, bb^ *P* value < 0.05 and 0.01, respectively, compared with the retention time of the single-dose treatment within each group.

Figure 3 showed that rats which were exposed to 12 h restraint stress and received vehicle decreased the retention time after a single dose of administration and both at 7 and at 14 days of treatment (*P* value < 0.05, 0.001, and 0.01, resp., compared with naïve control group). Tianeptine could mitigate the reduction of retention time in restraint rats (*P* value < 0.01 all, compared with vehicle + stress treated group). Interestingly, all doses of* N. nucifera* used in this study could mitigate the reduction of retention time in restraint rats (*P* value < 0.05 all, compared to vehicle + stress) at 14 days of experimental period. It was found that the increased treatment duration to 7 and 14 days enhanced the retention time in rats which received stress and low dose of* N. nucifera* extract more than the retention time of this treatment group which was observed after the single-dose treatment (*P* value < 0.05 all, compared with the retention time of the single-dose treatment within the same group). However, no significant difference change was observed regarding the effect of the low dose extract on retention time at 7 and 14 days of treatment. The rats subjected to stress and medium dose of* N. nucifera* also significantly increased the retention time when the treatment was prolonged further to 14 days of treatment (*P* value < 0.01, compared with the retention time of the single-dose treatment within the same group). No other effects of treatment duration on retention time were observed.

### 3.2. Effect of* N. nucifera* Flowers Extract on Serum Corticosterone Level

**Figure 4:**
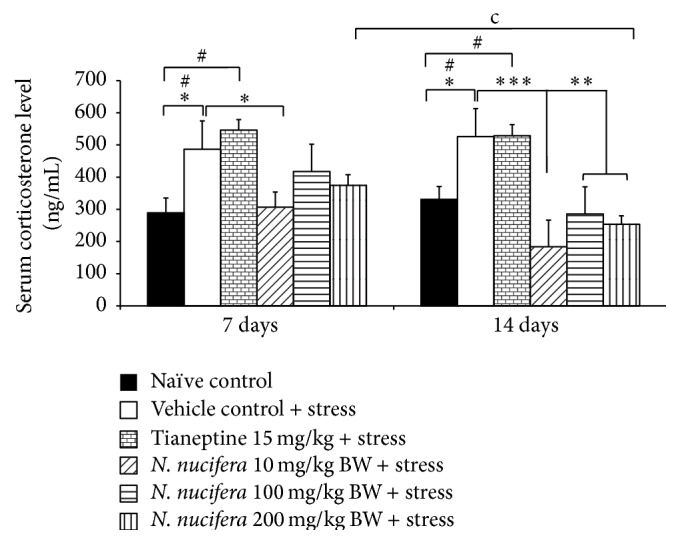
The effect of hydroalcoholic extracts of* N. nucifera* flowers extract on serum corticosterone levels of stress-exposed rats at 7 days and 14 days of treatment. Data were expressed as mean ± S.E.M. (*n* = 5/group). ^*∗*, *∗∗*, *∗∗∗*^ *P* value < 0.05, 0.01, and 0.001, respectively, compared with vehicle plus stress. ^#^ *P* value < 0.05, compared with control group. ^c^ *P* value < 0.05, compared with the serum corticosterone level at 7 days of treatment within each group.

Rats which received vehicle and repetitive stress exposure significantly increased serum corticosterone levels both at 7 and at 14 days of exposure time (*P* value < 0.05 all, compared to naïve control) as shown in Figure 4. Tianeptine failed to modulate the elevation of serum corticosterone in stress-exposed rats.* N. nucifera* flowers extract at low dose mitigated the elevation of serum corticosterone in stress-exposed rats at 7 and 14 days of exposure time (*P* value < 0.05 and 0.001, resp., compared to vehicle + stress). Stress-exposed rats which received the extract at doses of 100 and 200 mg·kg^−1^ BW significantly mitigated the elevation of this parameter when the exposure time was prolonged to 14 days (*P* value < 0.05 all, compared to vehicle + stress). In addition, paired *t*-test was also determined to evaluate the effect of treatment duration on corticosterone level. It was found that when the treatment was prolonged to 14 days of treatment, rats which received high dose of* N. nucifera* showed a significant reduction of serum corticosterone from that of the 7-day treatment (*P* value < 0.05, compared with 7 days within group). No significant differences were observed in other treatment groups.

### 3.3. Effect of* N. nucifera* Flowers Extract on Acetylcholinesterase Activity

**Figure 5:**
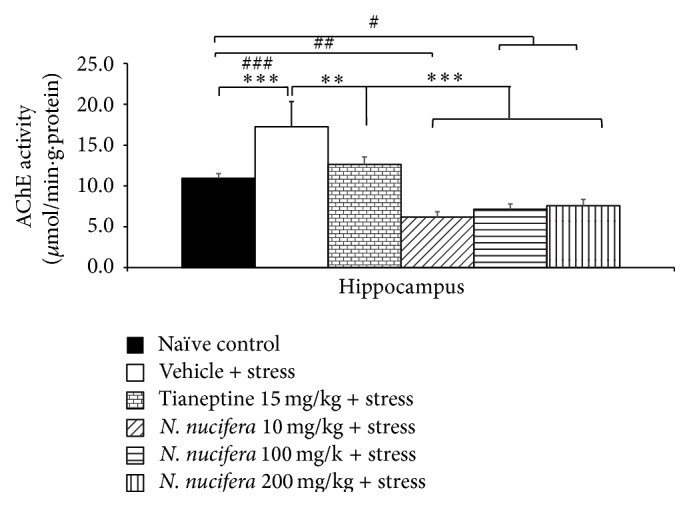
The effect of hydroalcoholic extracts of* N. nucifera* flowers extract on acetylcholinesterase activity in hippocampus of stress-exposed rats. Data were expressed as mean ± S.E.M. (*n* = 6/group). ^*∗∗*, *∗∗∗*^ *P* value < 0.01 and 0.001, respectively, compared with vehicle plus stress. ^#, ##, ###^ *P* value < 0.05, 0.01, and 0.001, compared with control group.

Figure 5 showed that rats subjected to restraint stress significantly increased AChE activity in hippocampus (*P* value < 0.001, compared to naïve control). Tianeptine attenuated the increased AChE activity in restraint stress rats (*P* value < 0.01, compared to vehicle + stress). It was found that* N. nucifera* flowers extract at all doses significantly decreased AChE activity in restraint stress rats (*P* value < 0.001 all, compared to vehicle + stress).

### 3.4. Effect of* N. nucifera* Flowers Extract on Monoamine Oxidase-A and Monoamine Oxidase-B

**Figure 6:**
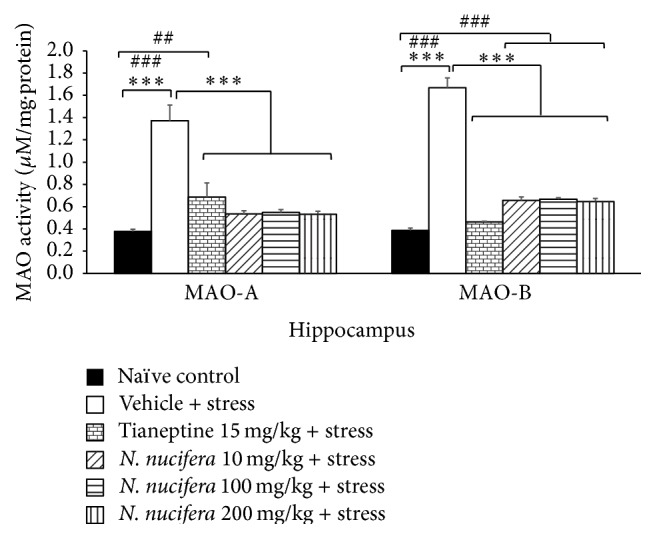
The effect of hydroalcoholic extracts of* N. nucifera* flowers extract on monoamine oxidase type A and monoamine oxidase type B of stress-exposed rats. Data were expressed as mean ± S.E.M. (*n* = 6/group). ^*∗∗∗*^ *P* value < 0.001, respectively, compared with vehicle plus stress. ^##, ###^ *P* value < 0.01 and 0.001, compared with control group.

Rats which received vehicle and were subjected to restraint stress significantly increased the activity of both MAO-A and MAO-B in hippocampus (*P* value < 0.001 all, compared to naïve control). Tianeptine mitigated the elevation of both MAO-A and MAO-B (*P* value < 0.001 all, compared to vehicle + stress). Rats that were subjected to restraint stress and received* N. nucifera* flowers extract at all doses significantly decreased MAO-A and MAO-B activities in hippocampus (*P* value < 0.001 all, compared to vehicle + stress) as shown in Figure 6.

### 3.5. Effect of* N. nucifera* Flowers Extract on Oxidative Stress Markers

Table 1 showed that repetitive restraint stress which received vehicle significantly decreased SOD, CAT, and GSH-Px activities but increased MDA level in hippocampus (*P* value < 0.001, 0.05, 0.001, and 0.001, resp., compared to naïve control group). Rats which received Tianeptine plus stress significantly increased SOD and GSH-Px activities but decreased MDA level in hippocampus (*P* value < 0.001 all, compared to vehicle + stress). All doses of* N. nucifera* significantly decreased MDA level in hippocampus (*P* value < 0.001 all, compared to vehicle + stress) but they failed to produce the significant changes on SOD and GSH-Px activities in hippocampus of stress-exposed rats. However, the medium and high doses of* N. nucifera* increased CAT activity (*P* value < 0.05 all, compared to vehicle + stress) in hippocampus of stress-exposed rats.

### 3.6. Effect of* N. nucifera* Flowers Extract on Survival Neurons in Hippocampus

**Figure 7:**
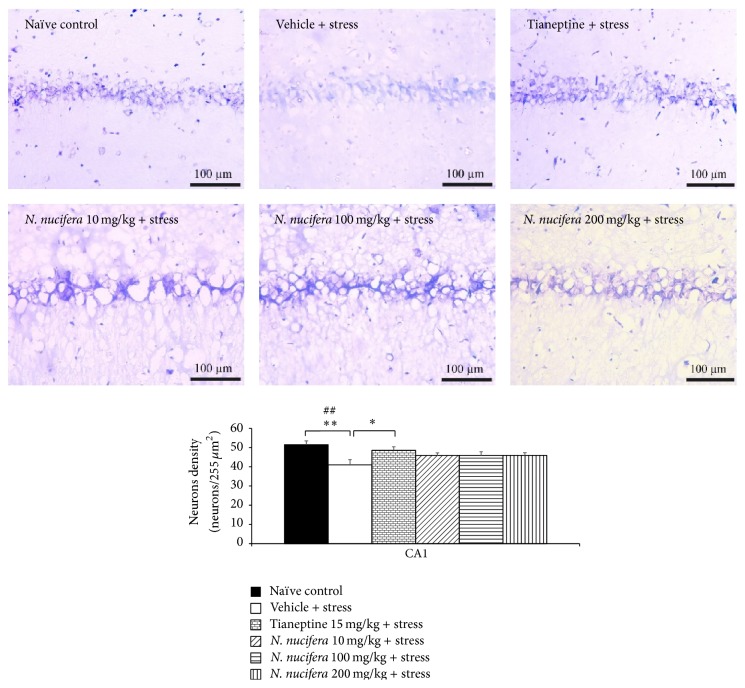
The effect of hydroalcoholic extracts of* N. nucifera* flowers extract on the density of survival neurons in CA1 of hippocampus of stress-exposed rats. Data were expressed as mean ± S.E.M. (*n* = 6/group). ^*∗*, *∗∗*^ *P* value < 0.05 and 0.01, respectively, compared with vehicle plus stress. ^##^ *P* value < 0.01, compared with control group.

**Figure 8 fig8:**
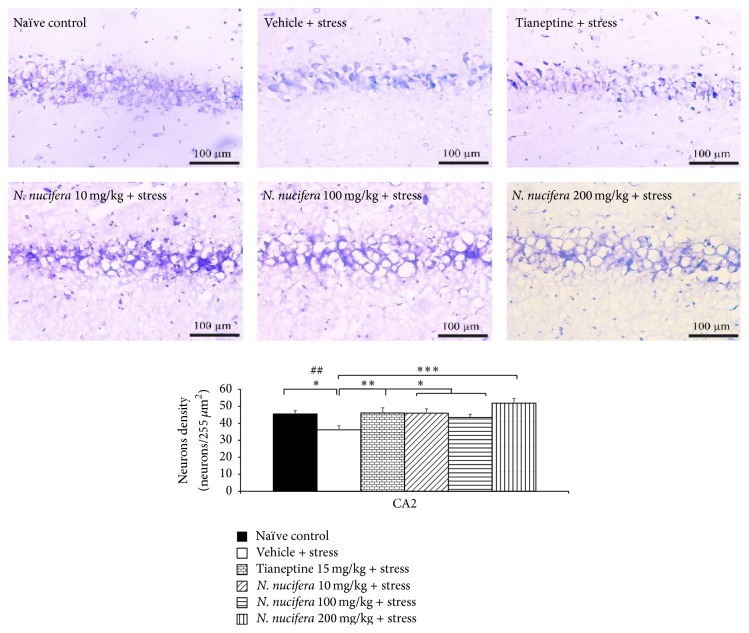
The effect of hydroalcoholic extracts of* N. nucifera* flowers extract on the density of survival neurons in CA2 of hippocampus of stress-exposed rats. Data were expressed as mean ± S.E.M. (*n* = 6/group). ^*∗*, *∗∗*, *∗∗∗*^ *P* value < 0.05, 0.01, and 0.001, respectively, compared with vehicle plus stress. ^##^ *P* value < 0.01, compared with control group.

**Figure 9 fig9:**
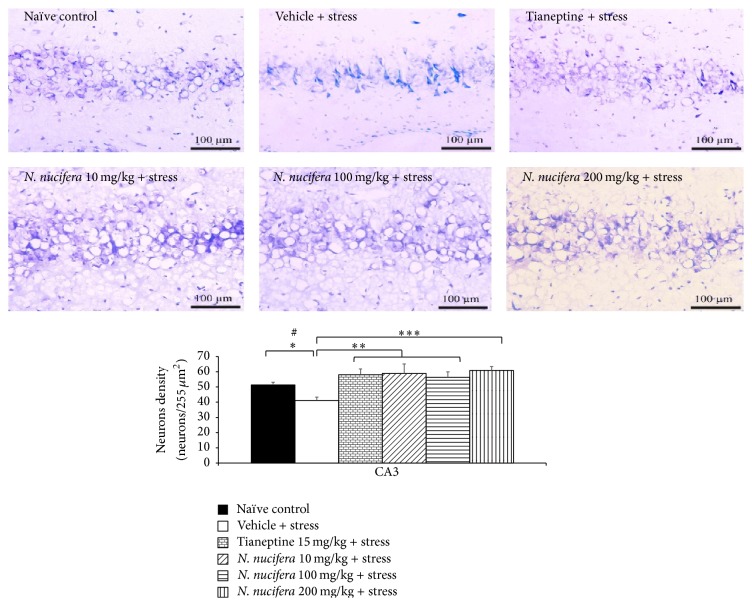
The effect of hydroalcoholic extracts of* N. nucifera* flowers extract on the density of survival neurons in CA3 of hippocampus of stress-exposed rats. Data were expressed as mean ± S.E.M. (*n* = 6/group). ^*∗*, *∗∗*, *∗∗∗*^ *P* value < 0.05, 0.01, and 0.001, respectively, compared with vehicle plus stress. ^#^ *P* value < 0.05, compared with control group.

**Figure 10:**
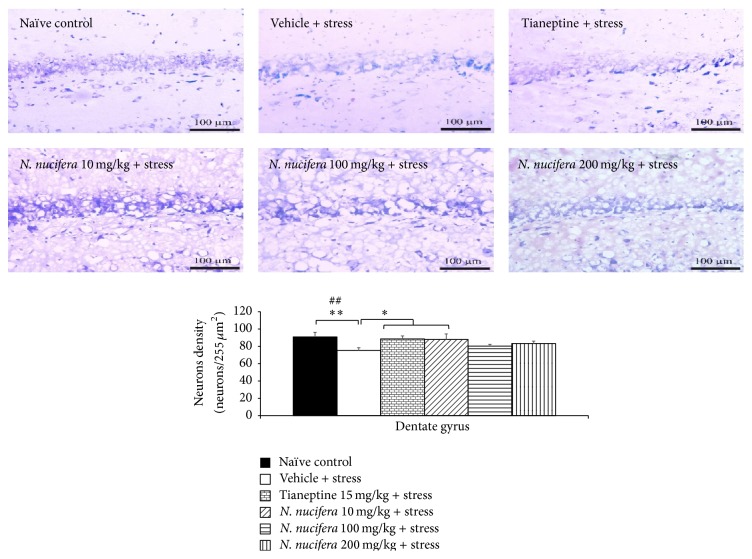
The effect of hydroalcoholic extracts of* N. nucifera* flowers extract on the density of survival neurons in dentate gyrus of hippocampus of stress-exposed rats. Data were expressed as mean ± S.E.M. (*n* = 6/group). ^*∗*, *∗∗*^ *P* value < 0.05 and 0.01, respectively, compared with vehicle plus stress. ^#^ *P* value < 0.05, compared with control group.

It was found that rats which obtained vehicle plus stress showed the decreased neurons density in CA1, CA2, CA3, and dentate gyrus (*P* value < 0.01, 0.01, 0.05, and 0.01, resp., compared to naïve control). Tianeptine increased neuron density in all areas mentioned earlier in stress-exposed rats (*P* value < 0.05, 0.01, 0.01, and 0.05, resp., compared to vehicle + stress). Interestingly,* N. nucifera* flowers extract at doses of 10, 100, and 200 mg/kg produced the significant increase in neurons density in CA2 (*P* value < 0.05, 0.05, and 0.001, resp., compared to vehicle + stress) and CA3 (*P* value < 0.01, 0.01, and 0.001, resp., compared to vehicle + stress) of stressed rats. Low dose of* N. nucifera* produced the significant increase of neurons density in dentate gyrus (*P* value < 0.05, compared to vehicle + stress) as shown in Figures 7 [Fig fig8] [Fig fig9]–10. No significant changes were observed in CA1 at all doses of* N. nucifera*.

### 3.7. Effect of* N. nucifera* Flowers Extract on Ki67 Proliferative Marker

**Figure 11:**
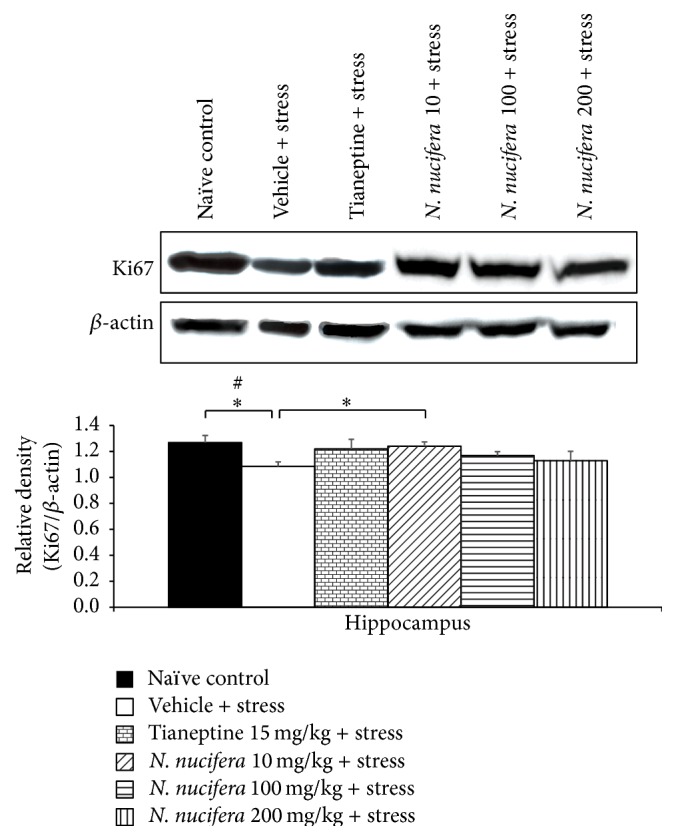
The effect of hydroalcoholic extracts of* N. nucifera* flowers extract on Ki67 proliferative marker of stress-exposed rats. Data were expressed as mean ± S.E.M. (*n* = 6/group). ^*∗*^ *P* value < 0.05, compared with vehicle plus stress. ^#^ *P* value < 0.05, compared with control group.

Figure 11 showed that repetitive exposure to restraint stress significantly decreased the level of Ki67, an adult neurogenesis marker, in hippocampus of stress-exposed rats (*P* value < 0.05, compared with naïve control). Tianeptine and* N. nucifera* extract at doses of 100 and 200 mg/kg failed to produce the change of Ki67 level in hippocampus of stress-exposed rats. However, stress-exposed rats which received* N. nucifera* extract at dose of 10 mg/kg could increase Ki67 level in the mentioned area (*P* value < 0.05, compared to vehicle + stress).

## 4. Discussion

It has been well known that hippocampus is a heterogeneous structure. Various subregions of hippocampus perform different functions. CA3 is preferentially involved in acquisition, consolidation, and retention phase of memory [19–21] whereas CA1 is involved in retention and retrieval of memory [19, 20] and dentate gyrus (DG) is important for encoding [20]. It has been recently shown that CA2 plays minor role in spatial memory but it plays an important role in social memory and social recognition [22]. In addition, hippocampus is also recognized as the special area at which neurogenesis can occur throughout adult lives [23]. Recent findings have shown that adult neurogenesis plays role in learning and memory [24].

Various processes of learning and memory depend not only on area specificity of hippocampus but also on various types of neurotransmitters. It has been shown that acetylcholine is essential for the encoding process, the first phase of memory, and dopamine plays the crucial role in both acquisition and retention of memory [25]. Norepinephrine is also implicated in hippocampus-based learning and memory especially acquisition process via adrenergic receptor [26].

The data obtained from this study demonstrated that the stress-exposed rats which received* N. nucifera* flowers extract at all doses used in this study showed the enhanced neuron density in CA2 and CA3 in hippocampus together with the improved spatial memory and the suppression effects of AChE, MAO-A, and MAO-B. On the basis of the information mentioned earlier, it has been suggested that the memory enhancing effect of* N. nucifera* flowers extract may occur partly via the increased neuron density in CA3 which in turn increases the acquisition, consolidation, and memory retention of memory leading to the improved spatial memory. In addition,* N. nucifera* flowers extract may also suppress AChE and both types of MAO resulting in the increased available ACh, NE, and DA in hippocampus which in turn increase encoding process and retention of memory and finally give rise to the improved memory.

In this study, the decreased serum corticosterone levels and the decreased oxidative stress status were also observed in rats that were subjected to 12 h immobilization stress and received* N. nucifera* flowers extract at all doses used in this study. The decreased serum corticosterone might increase the neurons density in hippocampus via the decreased excitotoxicity induced by corticosterone in hippocampus [27]. Besides the decreased serum corticosterone, the decreased oxidative stress in hippocampus also has important role in the increased density of survival neurons in the mentioned area. Although the oxidative stress status was very much improved, only the elevation of CAT activity was observed. Therefore, other factors such as the increased nonenzymatic antioxidant activity and the decreased oxidative stress formation might also have the role in the reduction of MDA level.* N. nucifera* flowers extract also increased Ki67 expression which in turn indicated the increased hippocampal neurogenesis in stress-exposed rats which received low dose (10 mg·kg^−1^ BW) of extract. Therefore, besides the mechanisms just mentioned, the increased neurons density in hippocampus in stress-exposed rats which received low dose of* N. nucifera* might be attributed to the increased adult neurogenesis in hippocampus.

The current study failed to show the dose dependent effect. The possible explanation might be attributed to the masking effect of other ingredients presented in the extract. In addition, the observed parameters were under the influence of many factors so no single relationship and dose dependent effect were observed. The effect of treatment duration was observed only on the change of retention time of the low dose treatment group but not in other groups. This might possibly occur because the change of retention time induced by the medium and high doses of extract had already achieved the maximum change and cannot produce any more change.

## 5. Conclusion

*N. nucifera* flowers extract was the potential neuroprotective and cognitive enhancer agent against stress-related brain damage and memory deficit. The possible underlying mechanisms are associated with the improved oxidative stress status, the increased adult neurogenesis, and the increased neurotransmitters which play the role in learning and memory such as acetylcholine, dopamine, and norepinephrine. They can also provide benefits as neuroprotectant and memory enhancer against stress-induced memory deficit. In addition, most of them are not expensive and easy to approach. Therefore, they may serve as the potential neuroprotective and memory enhancing agent. However, further researches concerning the active ingredient, pharmacokinetics, and drug interaction are essential before moving to the clinical trial.

## Figures and Tables

**Table 1:** Effect of *N*. *nucifera* leaves extract on oxidative stress markers (SOD, GSH-Px, CAT, and MDA).

Group	SOD	GSH-Px	CAT	MDA
	unit/mg·protein	unit/mg·protein	unit/mg·protein	nmol/mg·protein
Naïve control	2.6 ± 0.5^*∗∗∗*^	13.5 ± 0.6^*∗∗∗*^	38.1 ± 5.4^*∗*^	0.19 ± 0.02^*∗∗∗*^
Vehicle + stress	0.5 ± 0.1^###^	2.9 ± 0.2^###^	18.2 ± 5.5^#^	0.66 ± 0.08^###^
Tianeptine 15 mg/kg + stress	2.8 ± 0.3^*∗∗∗*^	13.5 ± 0.5^*∗∗∗*^	23.9 ± 5.3	0.23 ± 0.01^*∗∗∗*^
*N*. *nucifera* 10 mg/kg + stress	1.0 ± 0.5^##^	2.8 ± 0.1^###^	32.0 ± 5.6^*∗*^	0.09 ± 0.00^*∗∗∗*^
*N*. *nucifera* 100 mg/kg + stress	0.7 ± 0.2^##^	2.8 ± 0.2^###^	32.2 ± 2.1^*∗*^	0.09 ± 0.00^*∗∗∗*^
*N*. *nucifera* 200 mg/kg + stress	0.5 ± 0.2^###^	2.8 ± 0.1^###^	36.3 ± 3.3^*∗*^	0.09 ± 0.01^*∗∗∗*^

^*∗*,*∗∗∗*^ *P* < 0.05 and 0.001, respectively, compared to vehicle plus stress.

^#,##,###^ *P* < 0.05, 0.01, and 0.001, respectively, compared to naïve control.

## Abbreviations

*N. Nucifera*: * Nelumbo nucifera*
AChE: Acetylcholine esterase
MAO-A: Monoamine oxidase type A
MAO-B: Monoamine oxidase type B
GSH-Px: Glutathione peroxidase
SOD: Superoxide dismutase
CAT: Catalase
MDA: Malondialdehyde.
